# The impact of novelty and emotion on attention-related neuronal and pupil responses in children

**DOI:** 10.1016/j.dcn.2020.100766

**Published:** 2020-02-04

**Authors:** Carolina Bonmassar, Andreas Widmann, Nicole Wetzel

**Affiliations:** a Leibniz Institute for Neurobiology, Magdeburg, Germany; b University of Applied Sciences Magdeburg-Stendal, Germany; c Institute of Psychology, Leipzig University, Leipzig, Germany; d Center for Behavioral Brain Sciences, Magdeburg, Germany

**Keywords:** Pupillometry, ERPs, Auditory attention, Change detection, Development, Attentional orienting

## Abstract

Focusing on relevant and ignoring irrelevant information is essential for many learning processes. The present study investigated attention-related brain activity and pupil dilation responses, evoked by task-irrelevant emotional novel sounds. In the framework of current theories about the relation between attention and the locus coeruleus-norepinephrine (LC-NE) system, we simultaneously registered event-related potentials (ERPs) in the EEG and changes in pupil diameter (PDR).

Unexpected emotional negative and neutral environmental novel sounds were presented within a sequence of repeated standard sounds to 7–10-year-old children and to adults, while participants focused on a visual task.

Novel sounds evoked distinctive ERP components, reflecting attention processes and a biphasic PDR in both age groups. Amplitudes of the novel-minus-standard ERPs were increased in children compared to adults, indicating immature neuronal basis of auditory attention in middle childhood. Emotional versus neutral novel sounds evoked increased responses in the ERPs and in the PDR in both age groups. This demonstrates the increased impact of emotional sounds on attention mechanisms and indicates an advanced level of emotional information processing in children. The similar pattern of novel-related PDR and ERPs is conforming to recent theories, emphasizing the role of the LC-NE system in attention processes adding a developmental perspective.

## Highlights

• ERPs and pupil dilation show similar response patterns to emotional novel sounds.
• Auditory attention-related processes are immature in 7–10-year-old children.
• Emotional information of novel sounds is processed similarly in children and adults.
• Emotional novel sounds have a greater impact on attention mechanisms.

## 1 Introduction

Focused attention can be captured by an unexpected occurrence of new events even if they are not relevant for the task at hand. How attention control develops during childhood and which factors influence attention control, has not been fully researched. The present study aimed to investigate the neuronal basis of the underlying mechanisms for the orientation of attention and the evaluation of task-irrelevant events in middle childhood (7–10 years). Moreover, we focused on the impact of emotional information of novel sounds on attention processes in children. The analysis of emotion and novelty processing and their interaction is highly relevant to understand how children deal with new but unexpected emotional information. We applied a new approach and simultaneously registered EEG and pupil size in order to identify corresponding psychophysiological correlates of attention in the event-related potentials (ERPs) and in changes of the pupil diameter (Pupil Dilation Response; PDR). In children, pupillometry is easier to apply than neurophysiological or imaging techniques. Recent studies linked changes in pupil size to the activity of brain networks and their underlying cognitive functions (Eckstein et al., 2017). Therefore, our study is intended to provide a basis for future studies, focusing on the development of attention control, particularly with clinical and sensitive age groups. Knowledge about the development of attention processes can be used to improve learning environments and task structures, especially in schools (for an example, see Fisher et al., 2014).

### 1.1 Processing of unexpected and task-irrelevant sounds reflected by ERPs

**Fig. 1:**
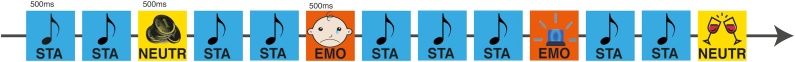
Sound sequence. Participants were instructed to ignore the oddball sound sequence and to focus on a silent video clip. Environmental sounds (EMO = emotional novel sounds; NEUTR = neutral novel sounds) were pseudo-randomly presented within a sequence of repeated standard sounds (STA). Examples of novel sounds are illustrated in the trial structure (chinking coins, a crying baby, a siren, toasting glasses, etc.). A total of 56 different novel sounds were presented with a randomized stimulus-onset asynchrony of 1800−2080 ms. Sounds were not relevant for the task (watching a video clip), but novel sounds were expected to capture attention.

A number of studies argue that attentional orienting and the further processing of task-irrelevant information can impair performance (distraction effect, for review see Escera et al., 2000). This has been studied experimentally using versions of the auditory oddball paradigm. Oddball paradigms include a sequence of repeated standard sounds and infrequently, randomly presented oddball sounds that differ in one or more features from standard sounds (Fig. 1). On a behavioral level, oddball sounds frequently cause impaired performance in the task at hand in adults (for review see Friedman et al., 2001) and in children (for review see Wetzel and Schröger, 2014). In children, this distraction effect decreases throughout early (until 6 years) and middle childhood (7–10 years, Wetzel et al., 2019). The further maturation of specific underlying neuronal mechanisms during middle and late childhood was described by neurophysiological and imaging studies (Olesen et al., 2007; Wetzel et al., 2006). This is in line with the maturational time course of the brain. It is assumed that distraction effects on a behavioral level are the sum of costs of the orienting of attention towards a new event and benefits of an increase of arousal, caused by the novel event (Masson and Bidet-Caulet, 2019; SanMiguel et al., 2010; Wetzel et al., 2012). In the EEG, novel oddball sounds evoke a sequence of components in event-related potentials (ERPs) in school age children and adults. In the present study we especially focused on a component of the P3 family occurring around 300 ms after a novel sound onset. The two subcomponents of this P3 were labeled as early and late P3a (Escera et al., 1998; Yago et al., 2003) or as P3a and novelty P3 (Barry et al., 2016; Friedman et al., 2001; Masson and Bidet-Caulet, 2019)^1^. The P3a wave is assumed to index orienting of attention and enhanced evaluation of oddball stimuli (Alho et al., 1997; Escera et al., 1998; Polich, 2007). The P3a was observed in children and adults for fronto-central brain areas (for review see Wetzel and Schröger, 2014; Polich, 2007). The latency of the P3a decreases with age (Riggins and Scott, 2019) while age-related differences in amplitudes were inconsistently reported (e.g., Čeponienė et al., 2004; Gumenyuk et al., 2004). In addition, we analyzed two other attention-related ERP components: the P2 and the late discriminative negativity (LDN). The P2 component, typically occurring around 200 ms (Gajewski et al., 2018) originates mainly from the secondary auditory cortex (Bosnyak et al., 2004; Mahajan and McArthur, 2012) and spreads to fronto-central areas (Ponton et al., 2000). The P2 is associated with early classification processes of stimuli as target and with inhibition mechanisms in order to protect against interference (for review see Crowley and Colrain, 2004). P2 peak latency is not associated with age-related change from childhood to adulthood (for review see, Wunderlich et al., 2006), while age-related effects on amplitudes of the P2 remain inconsistent (Wunderlich et al., 2006). A late negative ERP component, the LDN, has a fronto-central scalp distribution and is elicited by unexpected deviant sounds in children (Čeponienė et al., 2004). Some authors discussed that LDN reflects reorienting of attention after distraction (Čeponienė et al., 2004; Shestakova et al., 2003). Latency and amplitude of the LDN considerably decrease with age (Cheour et al., 2001; Horváth et al., 2009; Putkinen et al., 2012) and there is only a scarce number of reports on the LDN in adults (Cheour et al., 2001).

### 1.2 Processing of emotional unexpected and task-irrelevant sounds reflected by ERPs

The processing of emotional information is very important for humans, even if emotion is task-irrelevant. Humans are sensitive to emotional information and unable to fully ignore affective stimuli (Pessoa, 2005; Vuilleumier, 2005; Vuilleumier et al., 2001). Infants already respond with increased arousal to emotional events within the first 15 months of postnatal life (Geangu et al., 2011; Wetzel et al., 2016). In the auditory modality, 7–12-year-old children showed an ERP pattern to emotional speech prosody, that was similar to adults in amplitude and latency but shifted in time (Lindström et al., 2012). Only a few studies focused on the processing of auditory emotional non-linguistic stimuli and the developmental trajectory has been either fragmentary or inconsistently described in the literature. A recent study observed improvements in the categorization of non-linguistic affective vocal expressions in children aged between 5 and 17 years (Grosbras et al., 2018), indicating a long-lasting development of emotion processing. In contrast, few other studies reported matured emotional processing until the age of 5–8 years (visual modality, Leventon et al., 2014; Solomon et al., 2012). We are not aware of auditory oddball studies using environmental emotional oddball sounds with children. There are a few studies with adults that observed increased P3a amplitudes in response to emotional compared to neutral stimuli (Pakarinen et al., 2014; Thierry and Roberts, 2007; Widmann et al., 2018) and increased P2 amplitudes (Masson and Bidet-Caulet, 2019).

### 1.3 Attention networks, the role of the locus coeruleus-norepinephrine system and pupil size

In the following section we introduce the relation between attention networks and the activity of the locus coeruleus-norepinephrine (LC-NE) system and corresponding psychophysiological markers (ERPs and PDR). Attention mechanisms during unexpected events can be described in the context of influential attention models. The neuroanatomical model of attention control by Corbetta and Shulman (2002) describes two separate brain networks involved in top-down selection processes (dorsal frontoparietal network) and in the detection of unattended and behaviorally relevant stimuli (ventral frontoparietal network). When a behavioral relevant distractor occurs, the ventral network interrupts and resets ongoing activity (Corbetta et al., 2008). It has been argued that this process is modulated by the LC-NE system which releases norepinephrine over cortical areas (Corbetta et al., 2008). The LC-NE system is also considered part of the visual attention model by Posner (2008), that includes an alerting, orienting, and executive attention network. These networks and their interactions develop considerably between the ages of 6 and 12 years (Pozuelos et al., 2014). Based on the model of orienting response by Sokolov (1963), Näätänen (1992) developed an auditory attention model. This model postulated that the orienting towards unexpected novel stimuli comprises costs of orienting attention and benefits of an increased arousal level (see also, Masson and Bidet-Caulet, 2019; SanMiguel et al., 2010; Wetzel et al., 2012). Increased arousal is related to the LC-NE system (Aston-Jones and Cohen, 2005) and can facilitate several sensory, motor and cognitive processes (e.g., Kahneman, 1973). Task-relevant or motivationally significant stimuli (e.g. novel stimuli) can evoke a phasic activation of the LC-NE system (for review see e.g., Sara and Bouret, 2012). Animal studies (for review see, Aston-Jones and Cohen, 2005; Sara and Bouret, 2012; for an experiment see, Joshi et al., 2016) and recently human studies (Murphy, O’Connell, O’Sullivan, Robertson, & Balsters, 2014) demonstrated that activity in the LC is reflected by phasic changes in pupil diameter. In a visual oddball study, Murphy and colleagues reported a covariation of pupil size with BOLD activity in the LC during the presentation of a visual oddball sequence. In the auditory modality, rare and unexpected sounds caused a pupil dilation (Friedman et al., 1973; Widmann et al., 2018).

Previous studies investigated the influence of the LC-NE system on the attention-related P3a and on pupil dilation (Murphy, Robertson, Balsters, & O’Connell, 2011; for review see, Nieuwenhuis et al., 2011). The authors discuss the hypothesis of communal processes involved in attention due to projections from a medullary pathway. That is, P3a and pupil dilation might be influenced by the LC-NE system and share attention related processes, for example in the framework of novelty and emotional information.

Based upon the literature on immature attention control in children, we expected increased amplitudes of attention-related ERP components in response to novel sounds (relative to standard sounds) in children compared to adults (Čeponienė et al., 2004; Wetzel, 2015). Whether the pupil diameter is sensitive to these age-related changes in middle childhood was an open question as we were not aware of previous similar pupillary studies. Because of the significance of emotional events for humans, we hypothesized increased attentional-related brain activity (P3a, PDR) in response to emotional novel sounds compared to neutral novel sounds (Pakarinen et al., 2014; Thierry and Roberts, 2007; Widmann et al., 2018). Results in the literature were inconsistent and thus it is still an open question whether the impact of emotion on attention is similar for children and adults.

## 2 Materials and methods

### 2.1 Participants

65 participants took part in the experiment. One participant was excluded from further analysis because of very high impedance values (>50 kΩ). The data of 32 healthy children (*M*_age_ = 8;10 (years; months), range 7;4–10;3, 15 females, 3 left-handed) and 32 healthy adults (*M*_age_ = 26;6 years, range 18–36;4, 17 females, 3 left-handed) were used in the study. Participation was rewarded by a voucher for a local toy shop and a certificate (children, 7€/hour) or by money (adults, 7€/hour). All participants gave written consent (both children, parents and adults). Participants confirmed a normal or corrected-to-normal vision, no medication with effects on the nervous system, and no history of attention-related disorders. Handedness was measured with a shortened German version of the Oldfield Handedness Inventory (Oldfield, 1971). The project was approved by the local ethical committee.

### 2.2 Stimuli

*Auditory stimuli*. A total of 56 environmental sounds were collected from the database of a previous study (Max, Widmann, Kotz, Schröger, & Wetzel, 2015). This database consisted of a set of 210 auditory stimuli, collected from the International Affective Digitized Sounds study (IADS, Bradley and Laeng, 2007), by Hasting et al. (2010), and from other databases as described by Max et al. (2015). In the study by Max and colleagues, the novel sounds had been rated on a 9-point scale for valence (1 = unpleasant — 5 = neutral — 9 = pleasant) and arousal (1 = calm — 9 = arousing).

In the present study, sounds were allocated to two categories: 28 high arousing negative sounds (*M*_valence_ = 2.64; *M*_arousal_ = 6.60, for example an ambulance siren or a crying baby) and 28 moderately arousing neutral sounds (*M*_valence_ = 5.28; *M*_arousal_ = 4.77, for example chinking coins or toasting glasses). An independent samples *t*-test was performed revealing that sound categories significantly differed in valence (*t(54)* =−19.86, *p* < .001) and arousal (*t(54)* = 16.11, *p* < .001). The complex standard sound was comprised of sinusoids with a fundamental frequency of 500 Hz including the second and third harmonic attenuated by -3 and -6 dB, respectively. Sounds had a duration of 500ms including faded ends of 5ms. They were presented at a loudness of 66.5 dB SPL (measured with PAA3 PHONIC Handheld audio analyzer, Phonic Corporation, Taipei, Taiwan). Loudness of sounds was equalized with root mean square normalization.

*Visual stimuli*. To draw attention away from sounds the same silent animated cartoon was presented to all participants. The cartoon dealt with the story of a sheep’s adventures in a city. The cartoon was played continuously while the four sound blocks were presented. Thus, the cartoon was not repeated for every block and systematic effects of the video presentation on auditory processing were prevented. The video was displayed at the center of a screen with a size of 20 cm wide and 10.8 cm high (18.9° × 10.3° visual angle) on a grey background screen with a mean luminance of 2.9 cd/m^2^. The mean luminance of the movie was 53.1 cd/m^2^.

*Apparatus and Software*. The auditory stimuli were presented via loudspeakers (Bose Companion 2 series III Multimedia speaker system) located at the left and the right of the screen. The visual stimuli were presented on a 23.6 inch VIEWPixx/EEG display (VPixx Technologies Inc.) with a resolution of 1920 (H) × 1080 (V) and a refresh rate of 120 Hz. The distance from the participants eyes to the screen was approximately 60 cm. The experimental stimulation was presented via Psychtoolbox (Version 3.0.15, Kleiner et al., 2007) using Octave (Linux, Version 4.0.0).

### 2.3 Procedure

Participants were instructed to focus on a silent video clip and to ignore the presented oddball sound sequence, including unpleasant emotional and neutral novel sounds. No further task was performed during the experiment. Participants sat on a recliner chair in an acoustically attenuated and electromagnetically shielded cabin. Illuminance in the cabin was held constant at a level of 61.1 lx (measured with MAVOLUX 5032B USB, GOSSEN Foto- and Lichtmesstechnik GmbH, Nürnberg, Germany). Each of the four blocks started with a five-point eye-tracker calibration and validation procedure. A total of 280 sounds were presented per block with a randomized stimulus onset asynchrony (SOA, varying from 1800 to 2080 with 40 ms steps). In one block, 80 % of the trials consisted of a standard sound (224) and 20 % of a novel sound (56; Fig. 1). Half of the novel sounds were emotional sounds (28) and half were neutral sounds (28). The sound sequence was pseudo-randomized and unique for each participant. This ensures that changes in brightness in the video clip do not systematically vary with sound types. Each novel was followed by at least two standard sounds. A total of 896 standard sounds and 224 novel sounds (112 emotional, 112 neutral) were presented during the session. Each novel was repeated 4 times in total (once per block). Each block lasted 9 min.

### 2.4 EEG and pupil data recording

The electroencephalogram (EEG) was recorded at a sampling rate of 500 Hz from a 31 channel ActiChamp amplifier and a 31 active electrode Braincap (Brain Products GmbH, Gilching, Germany). The electrodes were placed according to the extended 10–20 system: Fp1, Fp2, F7, F3, Fz, F4, F8, FC5, FC1, FC2, FC6, T7, C3, Cz, C4, T8, CP5, CP1, CP2, CP6, P7, P3, Pz, P4, P8, Oz, and at the left (M1) and right (M2) mastoids. Three electrodes recording the horizontal and vertical electrooculogram (EOG) were positioned to the left and right of the outer canthi of the eyes and below the left eye. The reference electrode was placed at the tip of the nose.

The pupil diameter of both eyes was recorded with an infrared EyeLink Portable Duo eye-tracker (SR Research Ltd., Mississauga, Ontario, Canada). The eye tracking was set up in remote mode at a sampling rate of 500 Hz.

### 2.5 Data analyses

The first two standard trials per block and the two standard trials immediately following a deviant sound were removed from further analysis, because they could be affected by previous novel sound processing (Wetzel, 2015). Only corresponding identical trials from both ERP and pupil data were analyzed. That is, trials excluded from any, pupil or EEG data, were excluded from both types of analyses.

#### 2.5.1 EEG data processing

EEG data analysis was implemented with MATLAB software and the EEGLAB toolbox (Delorme and Makeig, 2004). The signal was filtered offline with a 0.1 Hz high-pass filter (Hamming windowed sinc FIR filter, order = 8250, transition band width = 0.2 Hz) and a 40 Hz lowpass filter (Hamming windowed sinc FIR filter, order = 166, transition band width =10 Hz, Widmann and Schröger, 2012; Widmann et al., 2015). The data were segmented into epochs of 1 s duration including a 0.2 s pre-stimulus baseline. The raw data was filtered with a 1 Hz high-pass filter (Hamming windowed sinc FIR filter, order = 8250, transition band width = 0.2 Hz) and 40 Hz lowpass filter. Independent component analysis (ICA) was applied on the filtered (1 Hz) raw data. Data were segmented in epochs with the same duration as the 0.1–40 Hz filtered data but not baseline corrected (Groppe et al., 2009). As suggested by Winkler et al. (2015), the obtained demixing matrix was applied to the 0.1–40 Hz filtered data. ICA components were classified by the ICLabel EEGLAB plug-in for automatic independent component (IC) classification, manually selected and pruned (Pion-Tonachini et al., 2019). Component rejection was restricted to typical eye ICA components, i.e. blinks, horizontal and vertical pre-saccadic spike potential, horizontal and vertical movements of the corneo-retinal dipole and blink/eyelid induced artifacts. On average 4.7 components per subject were eliminated (16 % of the total number of ICA components were rejected). Subsequently, trials with amplitude differences exceeding 150 μV were excluded from the analysis. Individual average ERPs were computed per participant and sound type. Grand-average waveforms were computed on the basis of individual averages (the number of included trials per condition and the mean of the ratio of excluded trials due to artifacts is described in the Supplementary Material, Table S1).

#### 2.5.2 Pupil data processing

Eye tracker pupil diameter digital counts were calibrated using the method suggested by Marchak and Steinhauer (2011) and converted to mm. Blinks and saccades were marked by the provided eye tracker event markers. Since partial blinks are not reported by the eye-tracker, an additional function was programmed, detecting those blinks from the smoothed velocity times series, i.e. pupil diameter changes exceeding 20 mm/s including a 50 ms pre-blink and a 100 ms post-blink interval were removed from further analysis (Merritt et al., 1994). Segmented data epochs of 2 s duration (including a 0.2 s pre-stimulus baseline) were baseline corrected by subtracting the mean amplitude of the baseline period from each epoch (Murphy et al., 2014; Widmann et al., 2018). Trials where at least one eye was closed or not recorded throughout the complete trial and data during blinks were excluded from averaging. Individual average PDRs were computed per participant and sound type from the mean of both eyes.

## 3 Statistical analysis

### 3.1 Principal component analyses (PCA)

Traditional ERP analyses suffer from two major, partly related problems: the relatively arbitrary definition of analysis time windows and the overlap of ERP components considerably biasing estimates of amplitude, latency, and location. We therefore applied a temporal PCA analysis (ERP PCA Toolkit MATLAB toolbox by Dien, 2010) to our data aiming (a) to identify the constituent components of the ERP and (b) provide dependent measures of these components for inferential testing to solve these problems (Dien, 2012). PCA belongs to the class of factor-analytic procedures using eigenvalue decomposition to extract linear combinations of variables (latent factors) accounting for patterns of covariance observed in the data, presumably due to ERP components (Dien and Frishkoff, 2005).

**Fig. 2:**
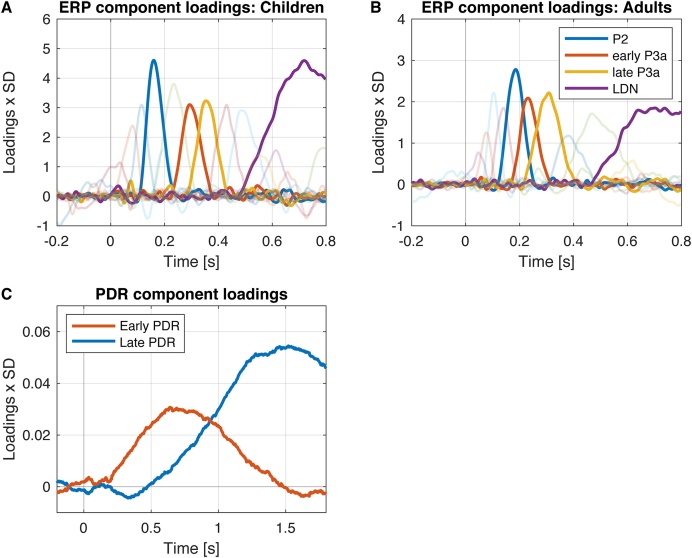
Scaled PCA component time courses (loadings * SD; μV) for ERPs (children Panel A, adults Panel B) and for PDR (both groups together, Panel C). Component loadings reflect the correlation of the variable (here time point) with the component (or factor). Component loadings do not reflect the amplitudes of the components. The scaling of the component loadings by the standard deviation illustrates the relative contribution of each component to the observed signal. Panel A and B: The amplitudes differences in the scaled loadings between age groups reflect the differences in variability across subjects, electrodes, and conditions between groups. The components of interest for children and adults are highlighted.

Temporal PCA results in a set of component loadings and a set of component scores. Component loadings reflect the strength of the association (correlation) of each variable (here time point) with each underlying factor and describe the time course of the components in temporal PCA. The component scores reflect the standardized weight with which each factor contributed to the observation, that is, combination of participant, condition, and electrode. Typically, components are sorted by the amount of variance they explain. Component loadings are frequently scaled by the standard deviation (SD) per variable (time point) to reflect real world units (here μV) and illustrate their relative amplitudes. Importantly, if the SD-scaled component loading vector (time course) is multiplied by the component score of an observation the result directly reflects the contribution of the component to the observed signal in μV units (see Dien, 1998, Appendix for a formal proof and Dien, 2012, for an accessible explanation; see Fig. 2). That is, the observed signal can be reconstructed as the sum of SD-scaled component loadings multiplied by component scores per observation.

PCA is particularly recommended for developmental populations reducing problems due to the enhanced noise level (Dien, 2012). In line with our study, recent studies on children used the temporal PCA (Kujawa et al., 2013; Speed et al., 2016). Other ERP-studies with adults report usage of temporal PCA (Foti et al., 2009; Kamp and Donchin, 2015; Widmann et al., 2018).

PCA was computed using Geomin rotation with ε = 0.5 (Scharf and Nestler, 2019), covariance relationship matrix and no weighting. The PCA analysis was conducted on the individual averages for all EEG channels (selection of electrodes or regions of interest for inferential analysis is a commonly applied procedure in temporal PCA, Hsu et al., 2014) and stimulus types (standard, emotional novel sound, neutral novel sound) separately for each group, because the component structure in the EEG differed between children and adults (for example the absence of a characteristic N1 component in children, see Wetzel et al., 2011). The number of components was determined using Horn’s parallel test. A total of 13 components were extracted from the ERPs in the adult group and 16 components from the ERPs of the children group. We focused our analyses on four components of interest, P2, early and late P3a, and LDN, related to inhibition or (re-) orienting of attention based on previous literature (Čeponienė et al., 2004; Escera et al., 2000; Wunderlich et al., 2006). We identified the respective components based on the typical time course and topography in the children’s and adults’ PCA. For analysis the following electrodes were selected on the basis of the literature where available (Čeponienė et al., 2004; Escera et al., 2000; Ruhnau et al., 2010; Wunderlich et al., 2006) and on the component peak across conditions otherwise: Cz (P2), Cz (eP3a), Fz (lP3a) and F4 (LDN). For an overview on the peak latencies and electrodes of components as well as the explained variance of the extracted components, see Supporting Material, Table S2.

We additionally analyzed component latencies as proposed in previous studies (Kiesel et al., 2008). We computed individual jack-knifing estimates for the component latencies separately for each group using an 80 %-relative peak amplitude criterion (Kiesel et al., 2008). In both groups the PCA was recomputed from 32 data subsamples leaving one subject out in each run. In each run the component loading (scaled by SD) corresponding to the components of interest was identified and the latency of the time point when the amplitude reached 80 % of the peak amplitude was measured. An 80 %-relative peak amplitude criterion was chosen as relative latency estimates have been shown to be less noisy than peak latency estimates using the jack-knifing technique (for detailed discussion see Kiesel et al., 2008). Individual latencies were retrieved from the subsample scores as suggested by Smulders (2010; Equation 1) to account for the reduced variance in the estimates due to the jack-knifing technique (equivalent to the adjustment of t/F-values suggested by Kiesel et al., 2008, Equation 1; the retrieval of individual latencies allowed straight-forward computation of Bayesian *t*-tests). The mean of the individual latencies was compared between groups using independent (Bayesian) *t*-tests.

The PDR revealed a biphasic pattern. The PCA was computed with the same parameters as for the ERPs but not separated by group (as the decompositions for both groups were highly similar if computed separately; see also Wetzel et al., 2016). Two components were extracted (see Fig. 2). The early peak presumably reflects the inhibition of the parasympathetic system (iris sphincter muscle relaxation) and the later peak presumably reflects the activation of the sympathetic system (iris dilator muscle contraction, Widmann et al., 2018).

### 3.2 Frequentist and Bayesian analyses

Statistical analysis was conducted using the software JASP (Version 0.9.1; JASP Team, 2017). As the PCA on the ERPs had to be computed separately for children and adults it was not possible to directly compare component scores between groups. We therefore computed component time courses per component, participant, electrode location, and condition by multiplying the component loading by the SD and by the component score. The resulting time course reflects the portion of the recorded waveform accounted for by each component scaled to μV (see Dien, 1998, for a proof), that is, comparable between the separate children and adult group PCA decompositions. The statistical analyses were based on the mean amplitude of this time course in the time window around the peak (+/- 20 ms) of every temporal component (in the component loadings). ERP difference amplitudes were obtained by subtracting the standard-related-ERP mean amplitude from the novel-related-ERP mean amplitude (Escera et al., 2000). For statistical tests of the difference amplitudes see Supplementary Material, Table S3.

ERP difference amplitudes were analyzed using frequentist and Bayesian repeated measures ANOVAs with the within subject factor emotion (emotional negative novel vs. neutral novel) and between subject factor group (children vs. adults). For the frequentist ANOVA an alpha-level of .05 was defined for all statistical tests and the η^2^ effect size measure is reported.

Bayes factors (*BF*_10_ and *BF*_Incl_ or “Baws Factor”, Mathôt, 2017) were estimated using 50,000 Monte-Carlo sampling iterations and a scaling factor r = 0.5 for fixed effects (corresponding to the default “medium” effect size prior for fixed effects in the R Bayes-Factor package, Morey et al., 2015) and r = 1 for the participant random effect (default “nuisance” prior for random effects in the R Bayes-Factor package). Data were interpreted as moderate evidence in favor of the alternative (or null) hypothesis if *BF*_10_ was larger than 3 (or lower than 0.33), or strong evidence if *BF*_10_ was larger than 10 (lower than 0.1, Lee and Wagenmakers, 2013). *BF_10_* between 0.33 and 3 are considered as weak evidence ("anecdotal evidence" following Lee and Wagenmakers, 2013). Interaction of factors were analysed using follow up ANOVAs (if more than three factors included) and *t*-tests (if two factors included; two-sided).

The pupil data were analyzed using frequentist and Bayesian repeated measures ANOVAs with the within subject factors emotion (emotional negative novel vs. neutral novel) and components (early component vs. late component) and between subject factor group (children vs. adults). The ANOVA was calculated directly on the PCA component scores (i.e., not re-scaled by *SD* and loadings as for the ERPs as we calculated the PCA on both groups together; see above).

## 4 Results

**Fig. 3:**
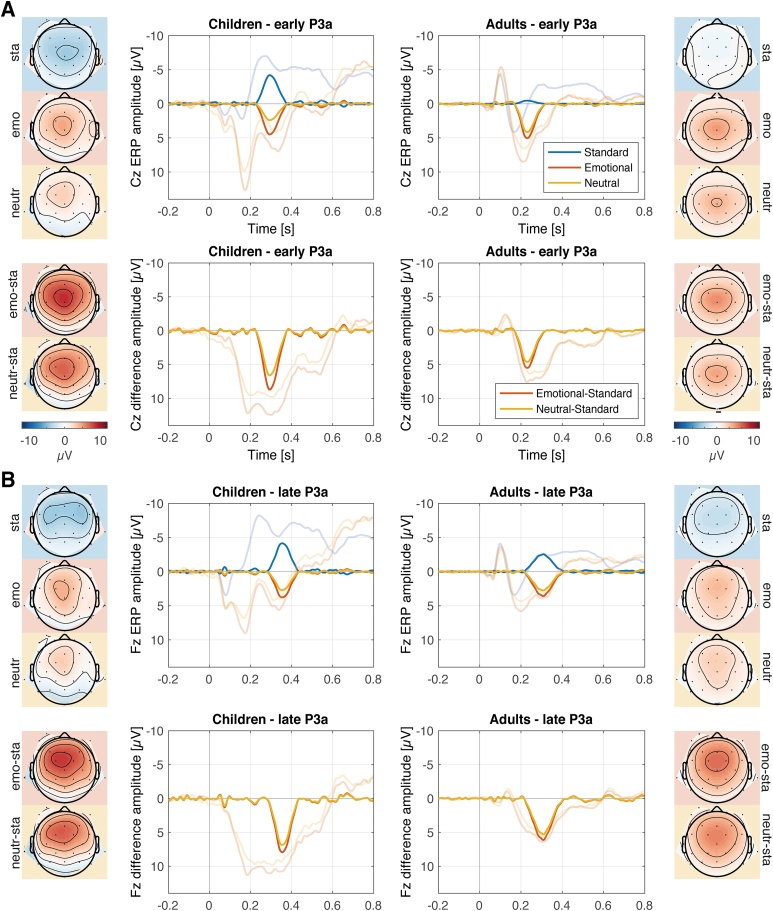
Panel A and B: PCA components (strong color) for the ERP components early P3a (A) and late P3a (B). The corresponding grand-averages at the specific electrode location are shown in transparent colors. The upper row of each Panel displays PCA components and ERPs evoked by standard sounds, emotional novel sounds, and neutral novel sounds. The lower row of each Panel displays the PCA components and ERPs of the difference waves of emotional novel minus standard and neutral novel minus standard. Topographies display the scalp distribution of the PCA components, on the left (children) and on the right (adults). Abbreviations at the side of the topographies indicate standard (sta), emotional (emo), neutral (neutr), and the difference waves emotional minus standard (emo-sta) and neutral minus standard (neutr-sta). Novel sounds evoked statistically significantly increased early and late P3a amplitudes in children than in adults. Emotional novel sounds statistically significantly evoked larger early and late P3a amplitudes than neutral novel sounds in both age groups.

**Fig. 4:**
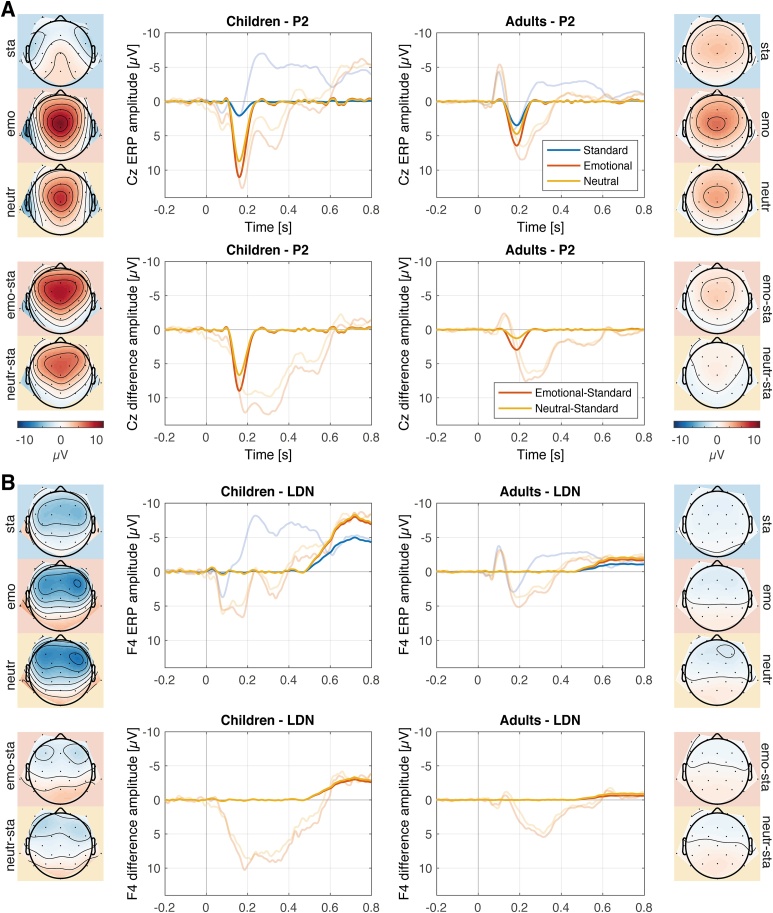
Panel A and B: PCA components (strong color) for the ERP components P2 (A) and LDN (B). The corresponding grand-averages at the specific electrode location are shown in transparent colors. The upper row of each Panel displays PCA components and ERPs evoked by standard sounds, emotional novel sounds, and neutral novel sounds. The lower row of each Panel displays the PCA components and ERPs of the difference waves of emotional novel minus standard and neutral novel minus standard. Topographies display the scalp distribution of the PCA components, on the left (children) and on the right (adults). Abbreviations at the side of the topographies indicate standard (sta), emotional (emo), neutral (neutr), and the difference waves emotional minus standard (diff-emo) and neutral minus standard (diff-neutr). Novel sounds evoked statistically significantly increased P2 amplitudes in children than in adults but not in the LDN. Emotional novel sounds statistically significantly evoked larger P2 amplitudes than neutral novel sounds in both age groups but not in the LDN.

**Fig. 5:**
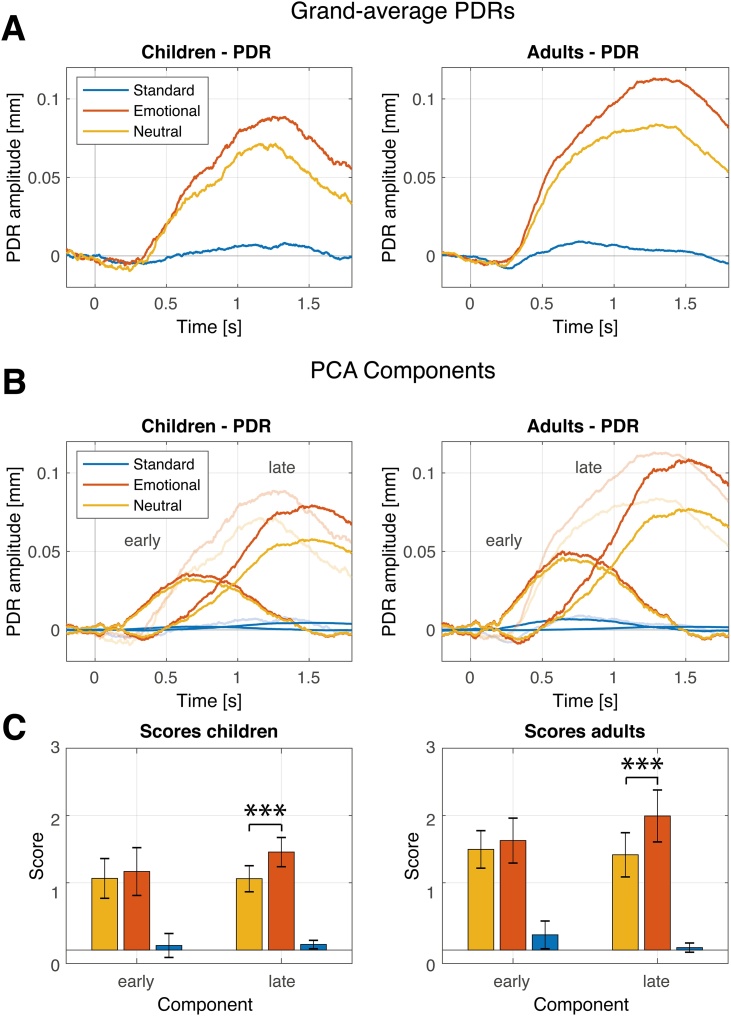
Panel A: Grand-average pupil dilation responses (PDRs) for emotional novel sounds, neutral novel sounds, and standard sounds for each age group. Novel sounds evoked statistically significantly increased PDRs compared to standard sounds in both groups. Panel B: Mean reconstructed component time courses (component loadings scaled by standard deviation (SD) and component scores per condition and age group; strong colors; mm) reflecting the portion of the recorded waveform accounted for by each component. The chronologically later component explains most variance and is discussed to reflect the activity of the sympathetic pathway of the autonomic nervous system. The earlier component is considered to reflect the activity of the parasympathetic pathway of the autonomic nervous system. The grand-average PDRs (transparent colors) were added for reasons of convenience and are identical to Panel A. Panel C displays mean PCA component scores (error bars show the 95 % confidence interval) that reflect the amplitudes for each sound type and group. Emotional novel sounds elicited statistically significantly increased amplitude for the late component only. Note. ***p < .001 (*t*-test).

The analyses were based on the difference amplitudes (novel-related-ERP mean amplitude minus standard-related-ERP mean amplitude, Escera et al., 2000) of the components P2, early and late P3a and LDN observed in both groups. A N2 component was pronounced only in children and was not included in the analysis (Fig. 3, Fig. 4, Fig. 5).

### 4.1 ERPs

#### 4.1.1 Early P3a

Early P3a peak latency was 230 ms in adults and 294 ms in children. The 80 %-relative peak latency of the early P3a components was significantly longer in children than in adults (275 vs. 211 ms; *t*(62) = 3.592, *p* < .001; *BF_10_* = 44.381). Early P3a was maximal over the vertex (Cz electrode) in both groups. The analysis of early P3a showed a main effect of the factor emotion (*F*(1,62) = 17.833, *p* < .001, η^2^ = .215), resulting from larger amplitudes in response to emotionally negative novel sounds, compared to neutral novel sounds. A main effect group (*F*(1,62) = 10.95, *p =* .002, η^2^ = .150) results from larger amplitudes in children compared to adults. No interaction of the factors emotion x group was observed (*F*(1,62) = 2.941, *p* = .091, η^2^ = .036). The Bayesian analysis provided strong evidence for the model including the main effects of emotion and group (*BF_10_* = 4411.044). The data do not provide conclusive evidence for or against an interaction effect of the factors emotion and group (*BF_Incl_* = 0.858).

#### 4.1.2 Late P3a

Late P3a peak latency was 308 ms in adults and 354 ms in children. The 80 %-relative peak latency of the late P3a components was significantly longer in children than in adults (333 vs. 277 ms; *t*(62) = 4.037, *p* < .001; *BF_10_* = 155.082). Late P3a was maximal over fronto-central electrode sites in both groups. The analysis of late P3a showed a main effect of the factor emotion (*F*(1,62) = 9.369, *p* = .003, *η^2^* = .131) resulting from larger amplitudes in response to emotionally negative novel sounds compared to neutral novel sounds. A main effect group (*F*(1,62) = 6.732, *p=* .012, *η^2^* = .098) results from larger amplitudes in children compared to adults. No interaction of the factors emotion x group was observed (*F*(1,62) = 0.173, *p* = .679, *η^2^* = .002). The Bayesian analysis provided strong evidence for the model, including the main effects of emotion and group (*BF_10_* = 46.769). The data provide moderate evidence against an interaction effect of the factors emotion and group (*BF_Incl_* = 0.282).

#### 4.1.3 P2

P2 peak latency was 186 ms in adults and 160 ms in children. P2 80 %-relative peak latency was not significantly different between children and adults (142 vs. 163 ms; *t*(62) = -0.656, *p* = .514; *BF_10_* = 0.307). P2 was maximal over the vertex (Cz electrode) in both groups. The analysis of P2 showed a main effect of the factor emotion (*F*(1,62) = 31.583, *p* < .001, *η^2^* = .335) resulting from larger amplitudes in response to emotionally negative novel sounds, compared to neutral novel sounds in both age groups. A main effect group (*F*(1,62) = 45.85, *p* < .001, *η^2^* = .425) results from increased amplitudes in children, compared to adults. No interaction of the factors emotion x group was observed (*F*(1,62) = 0.664, *p* = .418, *η ^2^* = .007). The Bayesian analysis provided strong evidence for the model including the main effects of emotion and group (*BF_10_* = 3.964 × 10^10^). The data provide weak evidence against an interaction effect of the factors emotion and group (*BF_Incl_* = 0.349).

#### 4.1.4 LDN

LDN peak latency was 702 ms in adults and 718 ms in children. LDN 80 %-relative peak latency was not significantly different between children and adults (643 vs. 607 ms; *t*(62) = 0.174, *p* = .863; *BF_10_* = 0.259). LDN was maximal over right frontal electrode sites (F4 electrode) in both groups. A main effect group (*F*(1,62) = 9.030, *p =* .004, *η^2^* = .127) was observed showing larger amplitudes in children, compared to adults. The LDN was not affected by emotion (*F*(1,62) = 0.791, *p =* .377, *η^2^* = .013). No interaction of the factors emotion x group was observed (*F*(1,62) = .001, *p* = .971, *η ^2^* = .000). The Bayesian analysis provided strong evidence for the model including the main effect group (*BF_10_* = 10.267). The data provide weak evidence against a main effect of the factor emotion (*BF_Incl_* = 0.390) and moderate evidence against an interaction effect of the factors emotion x group (*BF_Incl_* = 0.177).

### 4.2 Pupil diameter

#### 4.2.1 Pupil dilation response

The biphasic PDR to novel sounds was modulated by emotion and group. The early PDR peak latency was 640 ms while the late PDR peak latency was 1520 ms in both age groups. The analysis showed a main effect of the factor emotion (*F*(1,62) = 15.454, *p* < .001, *η^2^* = .198), resulting from larger pupil dilation in response to emotionally negative novel sounds, compared to neutral novel sounds. A main effect component (*F*(1,62) = 1.789, *p* = .186, *η^2^* = .028) was not observed. A main effect group (*F*(1,62) = 7.036, *p* = .010, *η^2^* = .102) results from larger pupil dilation in adults compared to children. No interaction of the factors emotion x group was observed (*F*(1,62) = 0.470, *p* = .495, *η^2^* = .006). A significant interaction of the factors emotion x component was observed (*F*(1,62) = 11.651, *p* = .001, *η^2^* = .157) resulting from larger pupil response for emotional novel sounds compared to neutral novel sound for the late (*t*(62) = 5.798, *p* < .001) but not for the early PDR component (*t*(62) = 1.140, *p* = .259). No interaction of the factors component x group was observed (*F*(1,62) < 0.001, *p* = .997, η^2^ < .001).

The Bayesian analysis revealed strong evidence for the model including the main effects emotion, component, group, and the interaction of the factors emotion and component (*BF_10_* = 326.562). The main effect component was not interpreted as the component scores are scaled differently and the factor was only included in the ANOVA to examine potential interaction effects. The data provide strong evidence for increased PDR amplitudes in response to emotional novel sounds, compared to neutral novel sounds on the late PDR component (Bayesian follow-up *t*-test emotional vs. neutral: *BF_10_* = 61242.266) and moderate evidence against an effect of emotion on the early PDR component (*BF_10_* = 0.254). The data provide moderate evidence against an interaction effect of emotion and group (*BF_Incl_* = 0.211) and an interaction effect of component and group (*BF_Incl_* = 0.195) and the three-way interaction of emotion, component and group (*BF_Incl_* = 0.378).

#### 4.2.2 Baseline mean pupil diameter

The observed baseline mean pupil diameter was 4.17 mm in the adult and 5.39 mm in the children group.

To separate growth-related differences in baseline pupil size from differences evoked by excitation, we estimated the expected baseline pupil diameter per participant, applying the implemented “Unified” model for light adapted pupil size (for details see Supplementary Material Part 1, Wheatley and Spitschan, 2018). While the expected baseline pupil was similar in children and adults, analysis indicated a significant difference between the expected and the observed baseline pupil in the children group only (for details see Supplementary Material Part 1).

## 5 Discussion

Task-irrelevant environmental novel sounds were presented in an oddball paradigm to 7–10-year-old children and adults while participants watched a silent video. Attention-related brain activity (EEG) and pupil dilation responses (PDR) were measured. Novel sounds per se and the emotional content of novel sounds caused increased amplitudes of attention-related ERPs (except for LDN in adults) and increased pupil diameter in both age groups (see Supplementary Material, Table S3). Results indicate enhanced processing of novel sounds that increased further when novel sounds contained emotional information.

Novel sounds evoked a characteristic pattern of ERP components containing P2, early and late P3a, and LDN, that have been associated with attention. These components were observed in the ERP difference waves computed from novel-ERPs minus standard-ERPs, which demonstrate different processing of novel sounds in relation to standard sounds. As these components partly overlap with each other we performed a PCA to separate components (Supplementary Material, Table S2). The PCA analysis revealed a structure of components that was not identical (for example an N2 component was pronounced in children only) but highly similar in children and adults. The temporal PCA could clearly classify and separate different components in time, therefore the selection of the components of interest for our study was fairly straightforward. This is a sustainable basis for comparing mechanisms associated to these components between age groups. In addition, two components in the pupil signal were extracted that were affected differently by the sounds’ novelty and emotional information.

### 5.1 Age effects on enhanced attention in response to novelty and emotion

Unexpected novel sounds and their emotional content caused pronounced ERP and PDR responses. On EEG level, both early and late P3a were observed in children and adults. Latencies were increased for both P3a components by 64 ms (early) and 46 ms (late) in children compared to adults, indicating slower attention processes in children. Amplitudes of the early and late P3a (difference wave novel-minus-standard) were increased in children compared to adults. Following the interpretation that P3a reflects orienting and evaluation (Escera et al., 2000), our results indicate immaturity of these processes in the presence of novel sounds in middle childhood. This interpretation is in line with previous findings on the long-lasting developmental trajectory of attention control, observed on a neuronal and behavioral level in the auditory modality (Horváth et al., 2009; Huotilainen et al., 2008; Ruhnau et al., 2010; Wetzel, 2015).

Emotional, compared to neutral, novel sounds evoked increased amplitudes of both P3a components, indicating enhanced attentional processing of emotional novel sounds. Results are in line with recent studies with adults reporting increased P3a amplitudes in response to emotional compared to neutral stimuli (Pakarinen et al., 2014; Thierry and Roberts, 2007; Widmann et al., 2018, but see, Czigler et al., 2007, who did not find an emotion effect). The orienting of attention towards emotional novel sounds and their enhanced evaluation was comparable between age groups. This indicates an advanced level of maturation of the involved emotion-related neuronal mechanism. These results are in line with findings that reported an early development of emotional processing until middle childhood (Leventon et al., 2014; Solomon et al., 2012).

In addition, novel sounds evoked a large transient pupil dilation compared to standard sounds in both age groups. Such transient changes in pupil size in response to oddball stimuli are related to the activity of the LC-NE system (Murphy et al., 2014). It has been shown in a fMRI study with adults that the novelty of visual oddball stimuli increased the activity in the LC (Krebs et al., 2018). The authors concluded that the noradrenergic system gives high priority to novel information. The significance of novelty for the LC-NE system was confirmed in a number of animal studies (Hervé-Minvielle and Sara, 1995; Larsen and Waters, 2018). The noradrenergic system is involved also in the processing of emotion (Berridge and Waterhouse, 2003; Nieuwenhuis et al., 2011; Ranganath and Rainer, 2003; Sara and Bouret, 2012). In line with these findings we observed increased PDR in response to emotional novel sounds. The sensitivity of the pupil to emotionally highly arousing pictures is long known (Hess and Polt, 1960) and was also observed in infants in response to the cry of a peer (Geangu et al., 2011; Wetzel et al., 2016). Novel sounds in the present study evoked a biphasic waveform that was separated by the PCA in two components. Steinhauer and Hakerem (1992) hypothesized that the two components might reflect the activity of the parasympathetic and the sympathetic pathways of the autonomic nervous system. They assumed that the chronologically early component reflects the inhibition of the parasympathetically controlled sphincter muscle and that the later component controlled the activation of the sympathetically innervated dilator muscle. As these muscles operate antagonistically, both result in pupil dilation. This hypothesis has been recently experimentally tested and results supported the hypothesis (see also, Bradley et al., 2008; Wetzel et al., 2016; Widmann et al., 2018). Therefore, the observed two components can be interpreted as indicators of the parasympathetic and the sympathetic activity of the autonomic nervous system (ANS). The emotional content of novel sounds further increased pupil diameter, but only for the later component. These findings are in line with recent studies with adults in the auditory and visual modality (Bradley et al., 2008; Widmann et al., 2018) and indicate that emotional arousal is reflected by the activity of the sympathetic nervous system (SNS). As suggested by Nieuwenhuis et al. (2011) and Murphy et al. (2011), the observed concurrent activation of P3a and the sympathetic component of the PDR supports the hypothesis of shared processes involved in attention due to projections from a common medullary pathway (Murphy et al., 2011; for review see, Nieuwenhuis et al., 2011).

As PDR components did not differ between children and adults, this concurrent activation in response to emotionally arousing sounds is suggested to function on a similar level in both age groups. In a previous study with infants, age effects in the interaction of both pathways of the ANS in response to highly arousing novel sounds were reported, indicating ongoing development (Wetzel et al., 2016). Even if the experimental details differ between the study by Wetzel et al. (2016) and our study, the lack of age differences in response to emotional novel sounds assumes a maturation of the underlying mechanisms during early childhood that has reached an advanced level in middle childhood.

The mean phasic pupil dilation response was reduced in children compared to adults. These unexpectedly reduced PDR amplitudes are not in line with the increased amplitudes in novel-related ERPs in children. A potential explanation for the observed differences between children and adults might be provided by systematic differences in tonic arousal. Decreasing phasic responses are expected with increasing tonic arousal and LC activity (Aston-Jones and Cohen, 2005; Gilzenrat et al., 2010; Kamp and Donchin, 2015). Additionally, higher tonic activity is reflected in larger baseline pupil diameters, limiting the dynamic range for pupil dilation (see e.g., Widmann et al., 2018, reporting larger PDRs in moderate compared to dark lighting conditions, note, in darkness the baseline pupil size is increased). We therefore tested for systematic differences in baseline pupil diameter between age groups. In fact, the observed baseline pupil diameter was considerably larger in children than in adults (by 29 %) and also considerably larger than predicted by a model considering age, luminance, and field of view (Watson and Yellott, 2012). The pupil size changes with age, following a U-shape with a maximal peak around 15–20 years (MacLachlan and Howland, 2002; Wilhelm, 2011). The model predicts almost identical mean pupil diameters due to the visual stimulation for both age groups (see Supplementary Material Part 1). While the predicted baseline pupil size was very precise in adults, children's observed baseline was larger than predicted (by 28 %). This indicates that the observed differences in the baseline pupil size resulted mainly not from physiological differences between age groups but from factors related to the experiment. For example, it is plausible that children were more excited by the video clip and the experimental situation. Another hypothesis might be that the children were more focused on the task compared to the adults. All hypotheses assume an increase in tonic arousal that is reflected in increased baseline pupil diameter and might affect the sound-evoked phasic PDR. As shown by Kamp and Donchin (2015) the negative effect of enhanced tonic arousal on phasic response amplitude is absent or much smaller for the P3 compared to pupil dilation. Further studies are needed to specify these relations in children.

### 5.2 Age effects on early and late processing of novelty and emotion

Early processing of novelty and emotion was related to the P2 component (Ponton et al., 2000). P2 amplitudes were increased in response to novel sounds compared to standard sounds indicating sensitivity of underlying processes for novelty. This novel-related increase was significantly larger in children than in adults indicating children's enhanced susceptibility to the novelty of sounds. Emotional novel sounds caused larger P2 amplitude than neutral novel sounds, demonstrating enhanced processing of the emotional information provided by novel sounds. This is consistent with previous literature focusing on adults. Increased amplitudes of P2, evoked by sounds with high valence ratings, were reported in adults (Masson and Bidet-Caulet, 2019). Similar to P3a results, the emotion effect did not differ between age groups. In line with previous studies, we observed no differences in latency between children and adults (for review see Wunderlich et al., 2006). The underlying mechanisms of the P2 are considered to provide the basis for subsequent cognitive processes. Some studies show that the P2 reflects stimulus classification processes (for review see Crowley and Colrain, 2004). Recently, Getzmann et al. (2018) interpreted larger P2 amplitudes in response to relevant, compared to irrelevant stimuli as classification of the target. Following this model, novel sounds and emotional novel sounds might be classified as highly significant, because they could require a behavioral response. This interpretation would be in line with studies reporting increased distraction effects on a behavioral level. Another hypothesis is that the P2 reflects inhibition processes (for review see Crowley and Colrain, 2004), that is, children less successfully inhibited the processing of task-irrelevant novel sounds or spent more resources on inhibition processes. Alho et al. (1987) observed, for example, an increased amplitude in the P2 component for non-target compared to target stimuli in an oddball paradigm. The authors interpreted this increase as increased effort in inhibition of non-target processing and protection against interference from irrelevant stimuli. Both hypotheses are in line with the long-lasting maturation of the prefrontal cortex, as this region is involved in novelty evaluation and inhibitory control (Case, 1992).

Late processing of novelty and emotion was associated with the late discriminative negativity component (LDN, Čeponienė et al., 2004). In children, LDN amplitudes were increased in response to novel sounds compared to standard sounds, indicating sensitivity for novelty. This is in line with previous findings and indicate a long developmental trajectory of attentional reorienting processes (Pearson and Lane, 1991). LDN has been hypothesized to reflect the processing of complex deviant sounds and can be observed in oddball paradigms requiring to ignore the sound sequence (Čeponienė et al., 2004; Cheour et al., 2001; Choudhury et al., 2015; Linnavalli et al., 2018). Increased LDN amplitudes might reflect increased effort to reorient the attention to the task at hand. In a dichotic listening task with 8 and 11-year-old children and adults (participants listened with one ear to targets and were signaled to switch attention to the other ear), increasing ability to reorient attention with age was reported by Pearson and Lane (1991). Gumenyuk et al. (2004, 2001) observed increased late negativity (LN) in younger children (8 years old) in comparison to older children (13 years old). This was interpreted to indicate the degree of attention engaged by the distracting sounds. Moreover, the authors observed a linear correlation between the reaction time (RT) and the LN, i.e. RT prolongation was correlated with increased amplitudes in LN. This was interpreted as increased effort for younger children in reorienting their attention back to the task. The age difference might be due to more intensive and prolonged processing of novel sounds in middle childhood, while adults were able to rapidly inhibit the processing of irrelevant events.

## 6 Limitation

Although we expected novel sounds to evoke increased amplitudes of P3a and PDR, P3a amplitudes were increased in children relative to adults while PDR amplitudes were reduced. We discussed the different pupil dilation as a result of the increased baseline pupil size in children. Future research is required to systematically investigate the relation of baseline pupil size in experimental conditions to phasic pupil dilation in dependence on age.

The increased amplitudes in the P3a might also be considered a consequence of the immature skull density and thickness in childhood. However, it is likely to only have a minor influence across development in the age groups tested in our study. Frodl et al. (2001) combined event-related potentials and magnetic resonance imaging (MRI) in an auditory oddball paradigm in order to investigate the influence of skull and scalp thickness on the ERP component P300. The authors observed no relation between P3a amplitude and fronto-central skull thickness and scalp thickness (Frodl et al., 2001). Maturational changes in EEG signals may be interpreted as a result of structural cortical modifications taking place in development. For example, gray matter volume decreases over childhood and adolescence and coincides with a reduction in the EEG power signal (Segalowitz et al., 2010). These maturational cortical modifications may be crucial when interpreting developmental changes in EEG activity.

## 7 Conclusion

Attention processes are modulated by the novelty and the emotional information of task-irrelevant sounds. Results of the present study indicate that involuntary attention in the presence of new events is still developing, while the emotional information is processed on an advanced level.

The use of pupillometry to investigate event-related attention mechanisms in children is a new and promising approach. We demonstrated that the phasic pupil dilation response reflects the processing of task-irrelevant novel sounds and their emotional content in children. The observed similar pattern of pupil dilation responses and well-known indicators of attention in the EEG allows conclusions on the neurophysiological and neuromodulatory interrelations of involved brain networks and their developmental pathways. Even if attention-related ERPs might be more sensitive to age-related changes in auditory attention processes, pupillometry can answer important questions on the development of attention, the activity of the ANS and the LC-NE system. This is particularly important for the investigation of sensitive age groups such as young children or atypically developing children. For example, it has been discussed that children diagnosed with ADHD suffer from instable or decreased brain arousal (Hegerl and Hensch, 2014), but that it might be normal for novel sound processing (Tegelbeckers et al., 2015; van Mourik et al., 2007). The present study provides the basis to investigate the interaction of these mechanisms using a method that is highly accepted by children.

## Declaration of Competing Interest

None.

## Footnotes

1 In the following we will term the early peak as early P3a and the late peak as late P3a. This does not endorse or reject models behind the labeling as this was not in the focus of the present study.
