# Supplementary material for: The impact of novelty and emotion on attention-related neuronal and pupil responses in children
Source: Dev Cogn Neurosci. 2020 Feb 4;42:100766. doi: 10.1016/j.dcn.2020.100766 (PMC7068055; doi:10.1016/j.dcn.2020.100766)
Supplement: Supplementary file 1 [file mmc1.pdf]

Supplementary Material

Table S1

Descriptive statistics (mean, SD, min, max) for the number of trials included in the individual ERP and the PDR averages per condition separately for children and adults (Panel A). The mean of the ratio of trials excluded due artifacts in the ERP data (voltage differences exceeding 150 µV per trial at any channel), artifacts in the pupil data (one or both eyes closed throughout the entire trial), or both within the same trial per condition (Panel B). Any trials excluded from analysis due to other reasons (first two standards per block, first two standards following a novel sound) are not included into the total number of trials used for the computation of the ratio of included/excluded trials. Note that trials with artifacts at either measure, ERP or pupil (or both) are excluded from all analyses. That is, the analyses of ERP and pupil data are based on corresponding trials. The ratio of the number of included trials to the number of total number of trials was significantly higher in adults than in children ($F(1,62) = 36.672$, $p < .001$, $BF_{Incl} = 37585$) and not significantly different between conditions ($F(2,124) = 0.978$, $p = .372$, $\varepsilon = .902$, $BF_{Incl} = 0.116$). The Bayesian ANOVA preferred the model including the group main effect only ($BF_{10} = 37698$).

	**Adults**				**Children**			
A Number of included trials:								
	**Mean (% total)**	**SD**	**Min**	**Max**	**Mean (% total)**	**SD**	**Min**	**Max**
**Standard**	421.2 (95.6 %)	36.1	300	446	342.3 (77.7 %)	79.6	76	437
**Emotional**	106.8 (96.0 %)	7.8	78	112	84.8 (76.1 %)	21.3	15	111
**Neutral**	107.0 (96.2 %)	8.5	77	112	85.4 (76.7 %)	20.8	16	111
B Mean of the ratio of excluded trials due to artifacts:								
		**ERP**	**Pupil**	**Both**		**ERP**	**Pupil**	**Both**
**Standard**		3.2 %	1.1 %	0.1 %		13.6 %	7.6 %	1.0 %
**Emotional**		2.8 %	1.0 %	0.1 %		15.1 %	7.4 %	1.3 %
**Neutral**		2.6 %	1.2 %	0.0 %		14.1 %	8.0 %	1.2 %

Table S2

Overview over the PCA components used for analysis (PCA temporal factor, first column). The chronological order of the factors reflects the proportion of explained variance (fifth column). For each component the corresponding latency (second column) and electrode (third column) is displayed. Corresponding ERP components to the PCA components are displayed in the fourth column.

	PCA temporal factor	Latency	Electrode	ERP Component	% variance explained
**Children**	1	718 ms	F4	LDN	45.1 %
	3	160 ms	Cz	P2	5.3 %
	5	354 ms	Fz	late P3a	2.7 %
	6	294 ms	Cz	early P3a	2.1 %
**Adults**	1	702 ms	F4	LDN	42.7 %
	2	186 ms	Cz	P2	24.6 %
	4	308 ms	Fz	late P3a	6.1 %
	5	230 ms	Cz	early P3a	3.1 %

Table S3

Frequentist and Bayesian paired *t*-test of the difference between emotional novel sound ERP and standard sound ERP (emo vs. sta) and neutral novel sound ERP and standard sound ERP (neutr vs. sta) for the ERP components P2, early P3a, late P3a, and LDN. All differences are statistically significant from zero (except for adult LDN emo vs. sta). Data were interpreted as moderate evidence in favor of the alternative (or null) hypothesis if BF_{10} was larger than 3 (or lower than 0.33), or strong evidence if BF_{10} was larger than 10 (lower than 0.1, Lee & Wagenmakers, 2013). BF_{10} between 0.33 and 3 are considered as weak evidence ("anecdotal evidence" following Lee and Wagenmakers, 2013).

		Children				Adults			
**Component**	**Condition**	***t***	***p***	***d***	BF_{10}	***t***	***p***	***d***	BF_{10}
**P2**	emo vs. sta	14.01	< .001	2.476	1.086×10^{12}	4.377	< .001	0.774	206.356
	neutr vs. sta	10.79	< .001	1.908	1.724×10^{9}	2.046	= .049	0.362	1.180
**Early P3a**	emo vs. sta	11.08	< .001	1.959	3.232×10^{9}	10.19	< .001	1.802	4.577×10^{8}
	neutr vs. sta	10.11	< .001	1.787	3.820×10^{8}	11.23	< .001	1.985	4.403×10^{9}
**Late P3a**	emo vs. sta	13.71	< .001	2.423	6.230×10^{11}	12.90	< .001	2.280	1.320×10^{11}
	neutr vs. sta	13.05	< .001	2.307	1.774×10^{11}	11.33	< .001	2.003	5.482×10^{9}
**LDN**	emo vs. sta	−3.494	= .001	−0.618	23.35	−1.926	= .063	−0.340	0.968
	neutr vs. sta	−4.679	< .001	−0.827	449.84	−3.329	= .002	−0.588	15.888

Part 1. Baseline mean pupil diameter and the "Unified" model

The observed baseline mean pupil diameter was 4.17 mm in the adult and 5.39 mm in the children group. We calculated the expected baseline pupil diameter per participant applying the "Unified" model for light adapted pupil size as suggested by Watson and Yellot (2012) and extended this to ages below 20 years (see, Watson & Yellott, 2012, Appendix 1) as implemented by Wheatley and Spitschan (Wheatley & Spitschan, 2018). As parameters we used the age of the participant in years, the

movie's mean luminance (53.1 cd/m^2) and visual angle (18.9°), and binocular viewing. The predicted pupil diameter was 4.24 mm for the adult and 4.22 mm for the children.

We compared the observed and the predicted baseline mean pupil diameter in an ANOVA with the factors, prediction error (observed vs. predicted) and group. It showed a significant interaction effect of prediction error and group ($F(1,62) = 53.7$, $p < .001$, $\eta^2 = .341$; and two spurious main effects prediction error and group). The corresponding Bayesian analysis also favored the model including both main effects and the interaction ($BF_{10} = 1.683 \times 10^{17}$). The observed baseline mean pupil diameter was not significantly different from the predicted pupil diameter in the adult group ($t(31) = -0.543$, $p = .591$, $d = -0.096$; $BF_{10} = 0.216$), but significantly larger than predicted in the children group ($t(31) = 11.41$, $p < .001$, $d = 2.017$; $BF_{10} = 6.520 \times 10^9$).

Moreover, we tested the pupil baseline between conditions and groups. The ANOVA including the factors condition (standard vs. emotional novel sound vs. neutral novel sound) and group showed a significant main effect of group ($F(1,62) = 48.12$, $p < .001$, $\eta^2 = .437$). The corresponding Bayesian analysis favored the model including the main effect group ($BF_{10} = 248884.022$). The data provide moderate evidence against a difference in baseline pupil diameter between conditions ($BF_{Incl} = 0.164$) and against an interaction effect of conditions and group ($BF_{Incl} = 0.233$).

References

Lee, M.D., Wagenmakers, E.-J., 2013. Bayesian cognitive codeling: A practical course. Cambridge University Press.

Watson, A.B., Yellott, J.I., 2012. A unified formula for light-adapted pupil size. J. Vis. 12 (10). doi:10.1167/12.10.12.

Wheatley, W., Spitschan, M., 2018. Watson & Yellott's (2012) unified formula for light-adapted pupil size [source code] Retrieved from https://github.com/spitschan/WatsonYellott2012_PupilSize/blob/master/wy_getPupilSize.m2018
